# A Subpopulation of Smooth Muscle Cells, Derived from Melanocyte-Competent Precursors, Prevents Patent Ductus Arteriosus

**DOI:** 10.1371/journal.pone.0053183

**Published:** 2013-01-31

**Authors:** Ichiro Yajima, Sophie Colombo, Isabel Puig, Delphine Champeval, Mayuko Kumasaka, Elodie Belloir, Jacky Bonaventure, Manuel Mark, Hiroaki Yamamoto, Mark M. Taketo, Philippe Choquet, Heather C. Etchevers, Friedrich Beermann, Véronique Delmas, Laurent Monassier, Lionel Larue

**Affiliations:** 1 Developmental Genetics of Melanocytes, Institut Curie, Orsay, France; 2 UMR_3347, CNRS, Orsay, France; 3 U1021, INSERM, Orsay, France; 4 Functional genomics and cancer, IGBMC, Illkirch, France; 5 Institute of Bio-Science and Technology, Nagahama University, Nagahama, Japan; 6 Department of pharmacology, Kyoto University, Kyoto, Japan; 7 Institut de Mécanique des Fluides et Solides, University of Strasbourg – CNRS, Strasbourg, France; 8 Medical Genetics and Functional Genomics, INSERM – UMR_S910, Marseille, France; 9 Aix Marseille University, Faculté de Médecine, Marseille, France; 10 School of Life Sciences, Ecole Polytechnique Fédérale de Lausanne (EPFL), Lausanne, Switzerland; 11 Laboratoire de Neurobiologie et Pharmacologie Cardiovasculaire – EA4438, Faculté de Médecine, Strasbourg, France; UT-Southwestern Med Ctr, United States of America

## Abstract

Patent ductus arteriosus is a life-threatening condition frequent in premature newborns but also present in some term infants. Current mouse models of this malformation generally lead to perinatal death, not reproducing the full phenotypic spectrum in humans, in whom genetic inheritance appears complex. The *ductus arteriosus* (DA), a temporary fetal vessel that bypasses the lungs by shunting the aortic arch to the pulmonary artery, is constituted by smooth muscle cells of distinct origins (SMC1 and SMC2) and many fewer melanocytes. To understand novel mechanisms preventing DA closure at birth, we evaluated the importance of cell fate specification in SMC that form the DA during embryonic development. Upon specific Tyr::Cre-driven activation of Wnt/β-catenin signaling at the time of cell fate specification, melanocytes replaced the SMC2 population of the DA, suggesting that SMC2 and melanocytes have a common precursor. The number of SMC1 in the DA remained similar to that in controls, but insufficient to allow full DA closure at birth. Thus, there was no cellular compensation by SMC1 for the loss of SMC2. Mice in which only melanocytes were genetically ablated after specification from their potential common precursor with SMC2, demonstrated that differentiated melanocytes themselves do not affect DA closure. Loss of the SMC2 population, independent of the presence of melanocytes, is therefore a cause of patent ductus arteriosus and premature death in the first months of life. Our results indicate that patent ductus arteriosus can result from the insufficient differentiation, proliferation, or contractility of a specific smooth muscle subpopulation that shares a common neural crest precursor with cardiovascular melanocytes.

## Introduction

The ductus arteriosus (DA) is a normal fetal artery linking the aortic arch to the left pulmonary artery in mammals (bilateral in birds), and allowing the blood to bypass the lungs *in utero* (reviewed in [1]). The blood circulation of amniotes then changes dramatically at birth. Once the lungs ventilate, blood flow reverses as pulmonary resistance decreases. The higher oxygen levels induce onset of DA closure through functional constriction of its muscular *tunica media*. This process initiates proliferation, migration, extracellular matrix production, and, through resultant hypoxia, the apoptosis of the cells forming the DA.

After birth, full closure of the DA can fail (patent ductus arteriosus or PDA), potentially leading to cardiac failure. In most cases, maintenance of an increased volume load to the left heart causes dilation of the left cardiac cavities associated with a progressive pulmonary vascular remodeling due to excess volume in the pulmonary artery bed. This compromises postnatal health by leading to respiratory complications such as pulmonary hypertension and edema. PDA is a frequent problem affecting premature children, with a prevalence greater than 40% in infants with a birth weight of 1.5 kg or less [2].

The final steps of DA closure include a drop in the levels of vasoactive hormones such as prostaglandin E2 (PGE2), which plays a critical role in maintaining the necessary open status of the DA during fetal development, followed by remodeling of the *tunica media* under the influences of hypoxia. In PDA due to prematurity, closure can usually be achieved by treatment with cyclooxygenase inhibitors such as indomethacin or ibuprofen, which block prostaglandin synthesis [3]. Such drug-induced closure is more difficult to obtain in cases of PDA in at-term newborns: the rate of success in these cases is only about 30% [3]. A properly closed DA ultimately undergoes physiological fibrosis to evolve into the *ligamentum arteriosum* (LigA).

In humans, mutations in *TFAP2B* (encoding the AP-2β transcription factor) and *MYH11* have been identified in cases of isolated, inherited PDA [4], [5], but most cases are believed to be multifactorial in origin and indeed often present as part of a syndromic spectrum, suggesting that the condition is associated with a complex genetic inheritance. A number of PDA mouse models have been produced, in which lack of DA closure generally leads to death within three days of birth (P3). Single gene mutations in *Hpgd*, *Ptger4*, *Ptgs2*, *Foxc1* or *Myh11* result in the absence of, or delay in, DA closure [6], [7], [8], [9], [10], [11]. In some cases, affected mice may be rescued by indomethacin injection [7]. Double mutant mice, in which *Cox-1* and *Cox-2* are disrupted, also exhibit PDA [12]. A deletion encompassing 24 genes between the *Dgcr2* and *Hira* loci on chromosome 16 leads to death with the presence of an open DA, as does a nested deletion comprising *Tbx1* and three other genes [13]. Deletion of the equivalent chromosomal region in humans is associated with the complex DiGeorge syndrome, of which PDA is a common feature [14], [15]. The somatic ablation of myocardin (*Myocd*), using Wnt1::Cre or Pax3::Cre mice, leads to the failure of DA closure [16]. These two promoters are active in early postmigratory neural crest cell derivatives, comprising the smooth muscle cells (SMC) of the DA.

The heart and its associated great arteries are composed for the most part of cells derived from the mesoderm, but with a critical contribution from neural crest cells [17]. Vagal neural crest cells (VNCC) can differentiate into numerous derivatives including neurons and glial cells of the enteric, autonomic and somatic nervous systems, SMC, mesenchymal cells and melanocytes (Mc). The *tunica media* of the DA, which like other pharyngeal arch-remodeled arteries consists mainly of SMC, is derived from these VNCC [18], [19], [20] [21], [22], [23].

Recently, Mc have been observed in the heart, in the valves and septa and in the DA/LigA [22], [24], [25], [26]. Mouse lines producing the Cre recombinase under the control of the tyrosinase promoter (Tyr::Cre) [27] have been used to show that the DA/LigA contains cells derived from VNCC: a very small number of Mc (pigmented and recombined cells), SMC2 (non-pigmented and recombined cells, estimated to constitute 10–20% of the DA/LigA cell population) and SMC1 (non-pigmented and non-recombined cells, estimated to make up to 80–90% of the DA/LigA cell population) [22]. The tyrosinase promoter [28] is transiently active around day 9 of gestation (E9) in Mc precursors, called melanoblasts (Mb) and SMC2 precursors of the DA [22].

Activation of Wnt/β-catenin signaling favors the Mc fate in multipotent progenitors [29], [30]. Here, we generated mice that produce a constitutively active form of β-catenin (ctnnb1Δex3) in cells having expressed tyrosinase, to both explore the SMC2/Mc bipotency of a subset of VNCC and to evaluate the anatomical consequences of cell fate alterations in the DA.

## Results

### Melanoblasts replace a subpopulation of smooth muscle cells in *ctnnb1Δex3* mice

**Figure 1:**
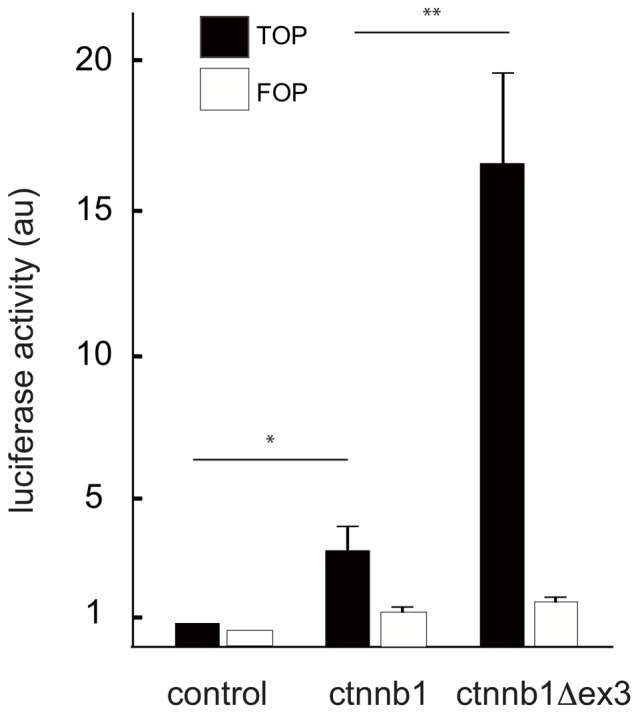
β-catenin (**ctnnb1**) **and ctnnb1Δex3 strongly activates the TOP promoter.** The empty, ctnnb1 and ctnnb1Δex3 expression vectors were co-transfected with TOP (black) and FOP (white) vectors in B16 melanoma cell lines [37]. Similar results were obtained with normal murine melanocytes (melan-a cells). The experiments were performed 3 times. *: p-value <0.05, **: p-value <0.01.

Recombinant *ctnnb1Δex3* mice were generated by producing an activated form of β-catenin in cells of the Tyr::Cre lineage. *LoxP* sequences had previously been inserted into introns 2 and 3 to flank the third exon of the gene encoding β-catenin (*ctnnb1*), for which the allele is also known as “f3” [31]. Exon 3 encodes serine and threonine residues involved in phosphorylation and degradation of the protein. The resulting modified β-catenin is reportedly more stable [32]. We verified *in vitro* that the deletion of exon 3 substantially increases protein activity (by five-fold) in B16 melanoma cell lines (**Figure** **1**), as has been demonstrated *in vivo* for recombined cells of the intestinal epithelium [31].

**Figure 2:**
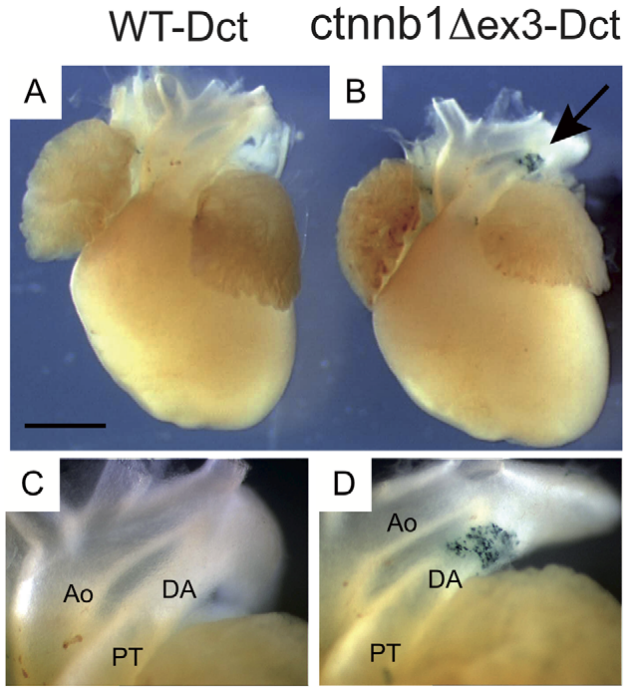
Melanoblasts are numerous in ctnnb1Δex3 DA. Ventral view of WT-Dct (A) and ctnnb1Δex3-Dct (B) E18.5 hearts stained with X-gal. Note that ctnnb1Δex3-Dct samples contain numerous β-galactosidase-stained cells (arrow) in the ductus arteriosus (DA). High magnification of the WT-Dct (C) and ctnnb1Δex3-Dct (D) DA regions, including the aorta (Ao) and the pulmonary trunk (PT). Scale bar (A, B)  = 1 mm.

**Figure 3:**
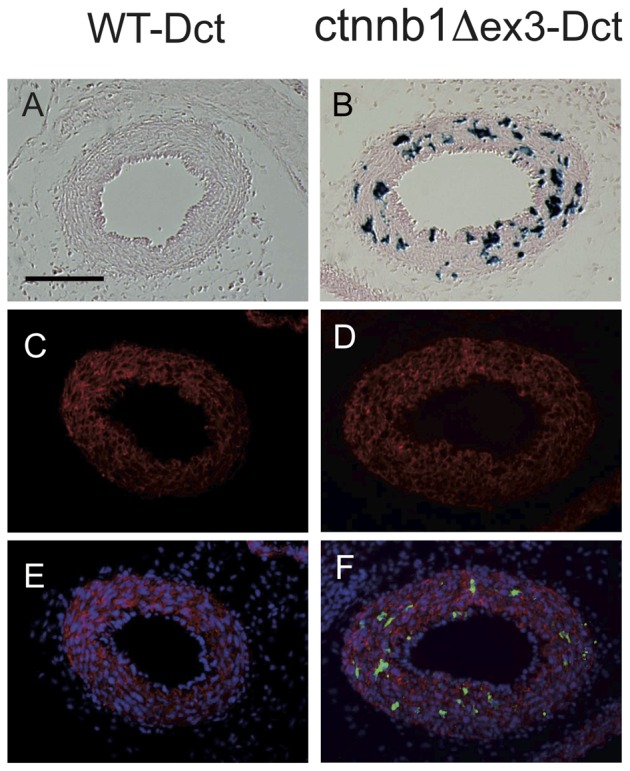
Transverse sections of WT and mutant ductus arteriosus. WT-Dct (A, C, E) and ctnnb1Δex3-Dct (B, D, F) DA. Sections were stained with X-gal (A, B), SMA (C, D) or DAPI in blue, SMA in red, β-galactosidase in green (E, F). Note that numerous cells producing β-galactosidase, corresponding to Mb, are present in ctnnb1Δex3-Dct DA (F). Genotypes: WT-Dct  =  *Tyr::Cre/*°; +/+; *Dct::LacZ/*°, ctnnb1Δex3-Dct  =  *Tyr::Cre/*°; *ctnnb1Δex3*/+; *Dct::LacZ/*°. Scale bar  = 100 µm.

Hemizygous *Tyr::Cre*/° mice were then crossed with heterozygous or homozygous floxed ( =  non-recombined) *ctnnb1Δex3*/+ or *ctnnb1Δex3*/*ctnnb1Δex3* mice to produce *Tyr::Cre*/°; +/+ (WT) and *Tyr::Cre*/°; *ctnnb1Δex3*/+ (ctnnb1Δex3) siblings (**Table** **S1**). These WT and ctnnb1Δex3 mice were initially backcrossed on a *Dct::LacZ* background, to visualize Mb in the DA. *Dct* is a member of the tyrosinase gene family and encodes the enzyme dopachrome tautomerase (also known as tyrosinase-related protein 2 or Trp2) involved in eumelanin synthesis. In *Dct::LacZ*/° reporter mice, the transgene *LacZ*, under the control of the *Dct* promoter, is expressed in Mb, retinal pigmented epithelium cells, and in the forebrain, but not in vascular smooth muscle cells. *Tyr::Cre/*°; +/+; *Dct::LacZ*/° ( =  WT-Dct) and *Tyr::Cre*/°; *ctnnb1Δex3*/+; *Dct::LacZ*/° ( =  ctnnb1Δex3-Dct) hearts were isolated, fixed and stained with X-gal on embryonic day (E)18.5. Whole-mount observation revealed that there were visibly more Mb in the mutant than the WT DA (**Figure** **2A–D**). Transverse sections confirmed that Mb were scarce in the WT-Dct DA, consistent with a previous study [22], but were numerous (60±23 Mb per section) in ctnnb1Δex3-Dct DA (**Figure** **3A, B**).

**Figure 4:**
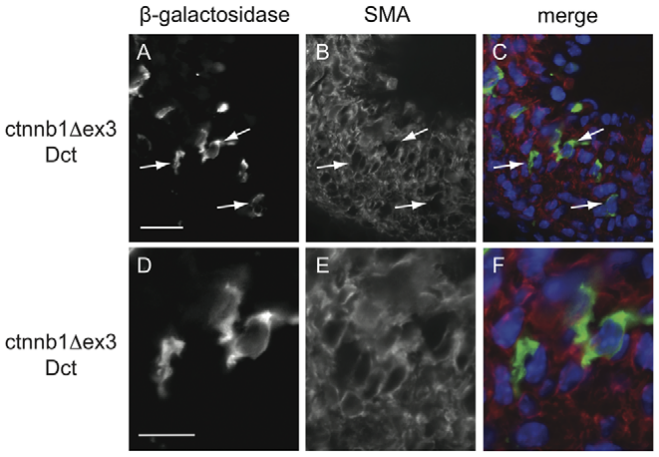
SMA-negative cells of the DA of ctnnb1Δex3-Dct mice are β-galactosidase-positive melanoblasts (**Mb**)**.** Low (A–C) and high (D–F) magnifications of transverse sections of ctnnb1Δex3-Dct DA stained with β-galactosidase (A, D in green corresponding to Mb) and SMA (B, E in red corresponding to SMC) antibodies. Superimposition of these two immunostainings includes DAPI in blue (C, F). Note that β-galactosidase-positive Mb (arrows) do not express SMA. These results strongly suggest that differentiated Mc do not have smooth muscle cell properties despite their common precursor. Nomenclature of the genotype: ctnnb1Δex3-Dct  =  *Tyr::Cre/*°; *f3/+*; *Dct::LacZ/*°. Scale bars (A)  = 20 µm, (D)  = 40 µm.

Antibodies directed against α-smooth muscle actin (SMA) and β-galactosidase were used for immunofluorescence analysis of E18.5 ctnnb1Δex3-Dct and WT-Dct DA (**Figure** **3C–F**). Expression of SMA and β-galactosidase was mutually exclusive: all β-galactosidase-positive cells were SMA-negative, and SMA-positive cells were β-galactosidase negative (**Figure** **4**). This suggested that at E18.5, the cells forming the DA were fully committed either to SMC (SMA-expressing) or to Mb (β-galactosidase-expressing due to the *Dct::LacZ* transgene).

**Figure 5:**
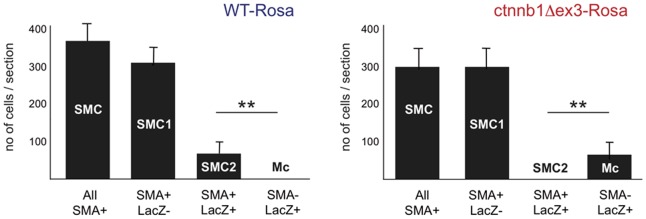
Melanoblasts replace a proportion of the smooth muscle cells in the ctnnb1Δex3 DA. SMA-positive and X-gal-positive cells in transverse sections of E18.5 WT-Rosa and ctnnb1Δex3-Rosa DA were counted. Three categories of cells were distinguished: non-recombined SMC1 (SMA+ LacZ−), recombined SMC2 (SMA+ LacZ+), and recombined non-SMC (SMA− LacZ+), corresponding to melanoblasts (Mb). Note that the number of SMA+ LacZ+ SMC2 in WT-Rosa DA is similar to the number of SMA− LacZ+ Mb in ctnnb1Δex3-Rosa DA. Genotypes: WT-Rosa  =  *Tyr::Cre/*°; *+/+*; *Rosa26/*°, ctnnb1Δex3-Rosa  =  *Tyr::Cre/*°; *ctnnb1Δex3/+*; *Rosa26/*°. Note, the production of a mutated form of β-catenin in recombined cells did not seem to greatly affect the number of floxed cells, suggesting that there was no cell non-autonomous effect on the floxed SMC. In both panels, there were significant differences between the numbers of SMA+, LacZ+ cells and SMA−, LacZ+ cells (for each genotype, the number of cells were estimated from 5–8 sections per embryo using 4 embryos: ** p-value <0.01).

To trace the progeny of recombined cells, we used the *Tyr::Cre* system for recombination and Rosa26R reporter mice for continuous β-galactosidase expression. *Tyr::Cre*/°; *ctnnb1Δex3*/+ (ctnnb1Δex3) or *Tyr::Cre*/°; +/+ (WT) mice were crossed with Rosa26R/+ mice to generate ctnnb1Δex3-Rosa (*Tyr::Cre*/°; *ctnnb1Δex3*/+; Rosa26R/+) and WT-Rosa (*Tyr::Cre/*°; +/+; *Rosa26R*/+) offspring. Immunostaining of the hearts and DA at E18.5 revealed the existence of three types of cells: (i) SMA-positive and β-galactosidase-negative cells ( = SMC1), (ii) SMA-positive and β-galactosidase-positive cells ( = SMC2) and (iii) SMA-negative and β-galactosidase-positive cells ( = Mb). The SMA-positive cells correspond to SMC, and the β-galactosidase-positive cells to recombined cells that had expressed the *Tyr::Cre* transgene. The numbers of SMC ( = SMC1+SMC2) were determined in transverse sections of WT-Rosa and ctnnb1Δex3-Rosa DA (**Figure** **5**). The total counts of SMC were higher in WT-Rosa (382±49) than in ctnnb1Δex3-Rosa (301±53) DA sections. However, the numbers of SMC1 were similar (300±65) in WT-Rosa and ctnnb1Δex3-Rosa, indicating that the number of SMC1 is not altered in ctnnb1Δex3-Rosa animals, but that SMC2 are lacking.

The numbers of SMC2+Mb per section were very similar in the DA of WT-Rosa and ctnnb1Δex3-Rosa (around 70) (**Figure** **5**). However, the numbers of SMC2 alone were 62±29 *versus* 5±3 per section in WT-Rosa and ctnnb1Δex3-Rosa, respectively, while the numbers of Mb were 5±5 versus 5,800±3,400 per 100 sections in WT-Rosa and ctnnb1Δex3-Rosa, respectively. These various observations show that on a *ctnnb1Δex3* background, Mb replaced most of the SMC2 in the DA. In other words, a subset of VNCC is bipotent for SMC2 and Mc, and responds to signaling through β-catenin.

### *ctnnb1Δex3* mice have a greatly dilated left atrium, which develops after birth

**Figure 6:**
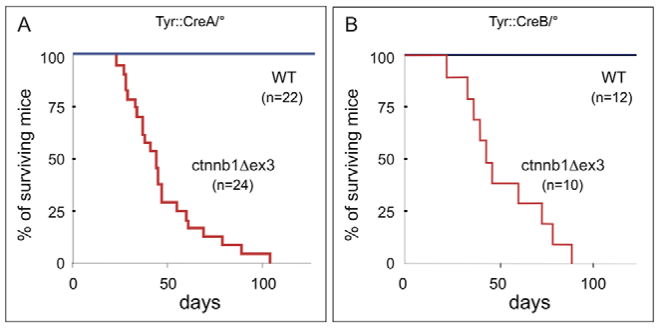
ctnnb1Δex3 mice die of heart failure between the second and fourth postnatal months. (A) Kaplan-Meier graph of survival of *Tyr::CreA/*°; *ctnnb1Δex3/*+ and WT littermate controls (*Tyr::CreA/*°; +/+ and °/°; *ctnnb1Δex3*/+). (B) Kaplan-Meier graph of survival of *Tyr::CreB*/°; *ctnnb1Δex3*/+ and WT littermate controls (*Tyr::CreB/*°; +/+). All members of both mutant populations perished before their fourth month of life, in contrast to the full survival of all their WT littermates.

As expected and according to specific crosses, *Tyr::Cre*/°; *ctnnb1Δex3/+* mice were produced in a Mendelian ratio (72 °/°; *ctnnb1Δex3/+* versus 67 *Tyr::Cre*/°; *ctnnb1Δex3/+* live births). However, all ctnnb1Δex3 mice died between 4 and 18 weeks of age (**Figure** **6A**). A distinct *Tyr::Cre* mouse line, *Tyr::CreB*, was used to see if the site of integration of the transgene or other cis-regulatory differences would affect the outcome [27]. Nonetheless, all *Tyr::CreB*/°; *ctnnb1Δex3*/+ mice died within a similar age range (between 3 and 12 postnatal weeks; **Figure** **6B**), indicating that the death of these animals was independent of the *Tyr::Cre* mouse line used.

**Figure 7:**
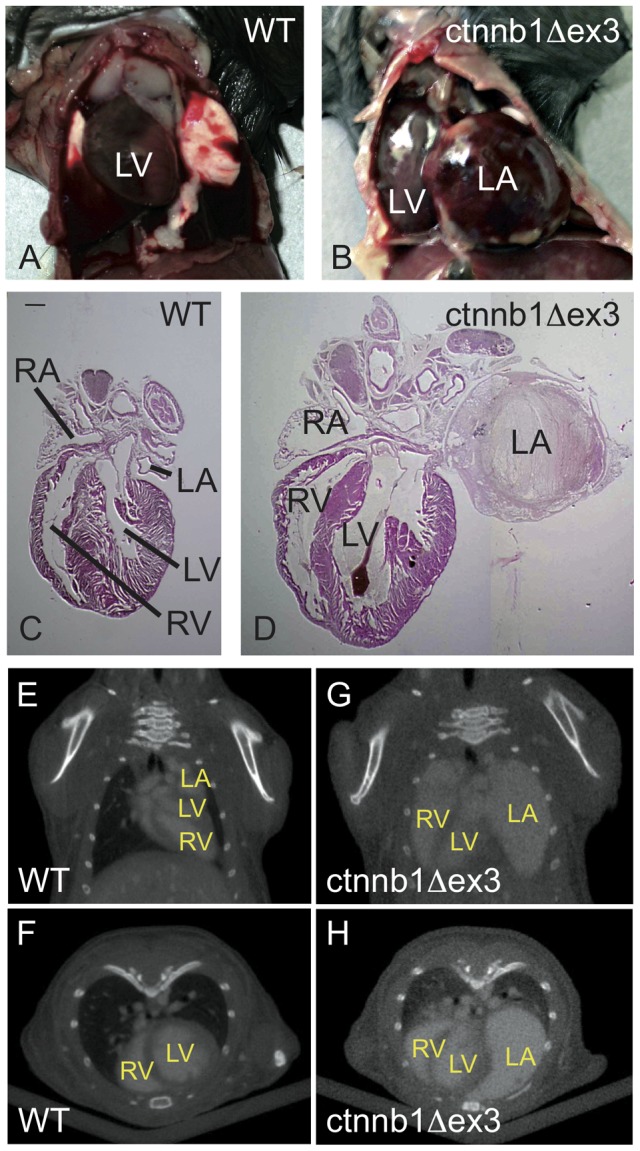
Dilatation of the ctnnb1Δex3 left atrium at postnatal day 28. Ventral views of *Tyr::Cre*/°; +/+ ( =  WT) (A) and *Tyr::Cre/*°; *ctnnb1Δex3*/+ ( =  ctnnb1Δex3) (B) open thoracic regions at postnatal day (P)28. Note the size of the ctnnb1Δex3 left atrium. Hematoxylin-eosin staining of WT (C) and ctnnb1Δex3 (D) sections. Note the fibrosis located in the mutant left atrium. Scale bar  = 1.5 mm. Frontal (E, G) and transverse (F, H) CT scan pictures of WT (E, F) and ctnnb1Δex3 (G, H) at the truncal level at P28. LA: left atrium; LV: left ventricle; RA: right atrium; RV: right ventricle.

The main clinical sign presented by ctnnb1Δex3 mice was prostration 24 hours prior to death. Such prostrated, heterozygous ctnnb1Δex3 mice and their wild-type siblings were sacrificed and autopsied. While never observed in the WT controls (**Figure** **7A, C**), a major dilation of the left atrium was observed macroscopically in all mutant mice (**Figure** **7B, D**) and a lesser dilation of the left ventricle was observed in about half of the mice (**Figure** **7D**), Micro-computed tomography (CT) analysis after Fenestra® injection was performed on WT and ctnnb1Δex3 mice (**Figure** **7E–H** **and movies S1, S2**). This revealed a rightward shift of the whole heart in live mutant animals, with a clear dilation of the left atrium (**Figure** **7G, H**).

**Figure 8:**
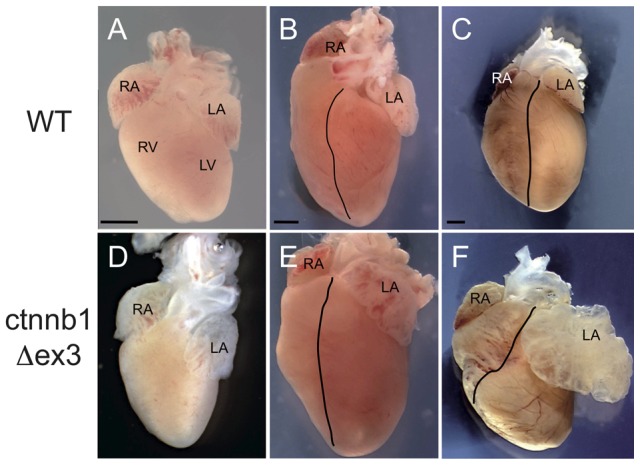
Progressive dilatation of the ctnnb1Δex3 left atrium during the first weeks of life. The *Tyr::Cre/*°; *ctnnb1Δex3*/+ ( =  ctnnb1Δex3) left atrium expands during the first postnatal month. Isolated WT (A–C) and ctnnb1Δex3 (D–F) hearts: A and D at postnatal day (P) 1, B and E at P10, C and F at P28. Scale bars, (A, D)  = 0.5 mm, (B, E)  = 1 mm, (C, F)  = 2 mm. Note that the ctnnb1Δex3 left atrium is abnormally large after P10. LA: left atrium, LV: left ventricle, RA: right atrium, RV: right ventricle.

WT and ctnnb1Δex3 hearts were examined at P1, P10 and P28. At P1, WT and ctnnb1Δex3 hearts were comparable, indicating that the enlargement of the left atrium was not associated with a congenital malformation due to a developmental defect (**Figure** **8A, D**). At P10, the ctnnb1Δex3 left atrium was visibly dilated (**Figure** **8B, E**). By P28, the expansion of the left atrium was substantial (**Figure** **7B, D** **and** **Figure** **8C, F**). Some dilation of the left ventricle, not hypertrophy, was also visible in a limited number of mutants by P28 (**Figure** **7D** **and** **Figure** **8F**).

**Figure 9:**
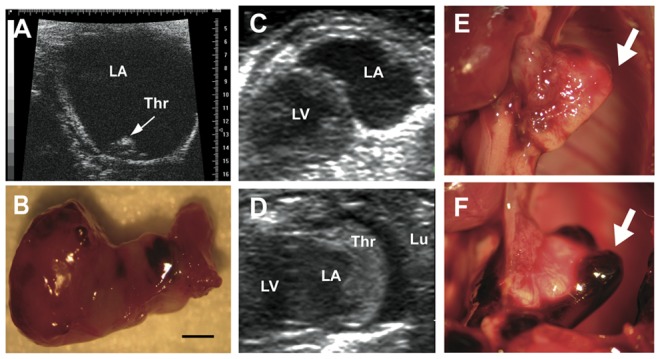
Thrombosis develops in mutant mice during the second postnatal month. (A) Ultrasound analysis of a *Tyr::Cre*; *ctnnb1Δex3*/+ ( =  ctnnb1Δex3) mouse with dilated left atrium containing a thrombus. (B) Isolated thrombus. Expansion of the left atrium is observed prior to the appearance of thrombosis in such ctnnb1Δex3 mice: (C) at 6 weeks of age and (D) at 8 weeks, from the same animal. *In situ* thrombus located in a ctnnb1Δex3 left atrium prior to (E) and after dissection (F). Thr: (thrombus), LA: (left atrium), LV: (left ventricle) and Lu: (lung). Scale bar, B = 1 mm.

Echocardiographic examination also showed substantial enlargement of the left atrium, associated with thrombi of various sizes (**Figure** **9**). Thrombus formation was subsequent to chamber dilation and aggravated over time (**Figure** **9C, D**). In one case, atrial myocardial rupture and pericardial blood effusion was observed on ultrasound analysis, and led to the death of the mouse. Atrial dilation was unlikely to be due to mitral valve dysfunction, because the Doppler mitral inflow pattern was normal (data not shown). Similarly, left-ventricular failure was not the cause, because the fractional shortening was normal. These data led to the hypothesis that death was due to the accumulation of large thrombi in the left atrium and/or its rupture.

### The ductus arteriosus is not fully closed in *ctnnb1Δex3* mice

**Figure 10:**
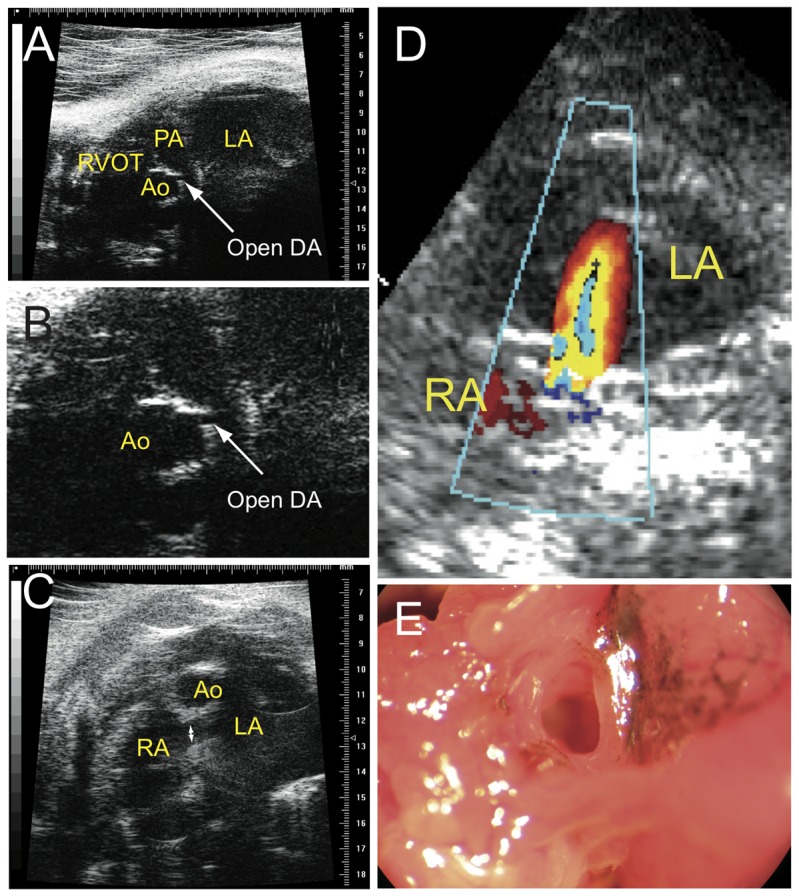
Abnormal circulation of the blood in ctnnb1Δex3 adult heart. (A) Ultrasound analysis showing an open *Tyr::Cre; ctnnb1Δex3*/+ (ctnnb1Δex3) DA. (B) Enlargement of (A) showing the DA. (C) Ultrasound analysis of a ctnnb1Δex3 patent foramen ovale. (D) Shunting through the foramen ovale as observed by echo Doppler analysis in ctnnb1Δex3 mice. (E) The foramen ovale remains open in ctnnb1Δex3 hearts. Note the presence of pigmented cells. LA: left atrium, RA: right atrium, Ao: aorta, RVOT: right ventricle outflow tract, DA: ductus arteriosus.

When the DA does not fully close after birth, a part of the systolic left-ventricular stroke volume goes directly into the pulmonary artery (left-to-right shunting), leading to a progressive overload of the pulmonary circulation by an increase in pulmonary pressure. Simultaneously, this volume overload triggers the progressive dilation of the left cardiac cavities. Surprisingly, in ctnnb1Δex3 mutant mice, this enlargement affected essentially the left atrium, the left ventricle being more modestly and not systematically dilated. Ultrasound analysis demonstrated that the DA remained open in postnatal ctnnb1Δex3 mice, which is never the case in WT mice (**Figure** **10A, B**). Ultrasound (**Figure** **10C**) and color Doppler analyses (**Figure** **10D**) showed blood flow back through the patent foramen ovale (**Figure** **10E**) from the right to the left atrium of all ctnnb1Δex3 mice, but not WT mice.

The death of the ctnnb1Δex3 mice thus seemed to result from the failure of DA closure and increased pulmonary pressure, leading ultimately to retrograde blood flow through the foramen ovale from the right to the left, driving the progressive dilation of the left atrium and thrombus formation.

**Figure 11:**
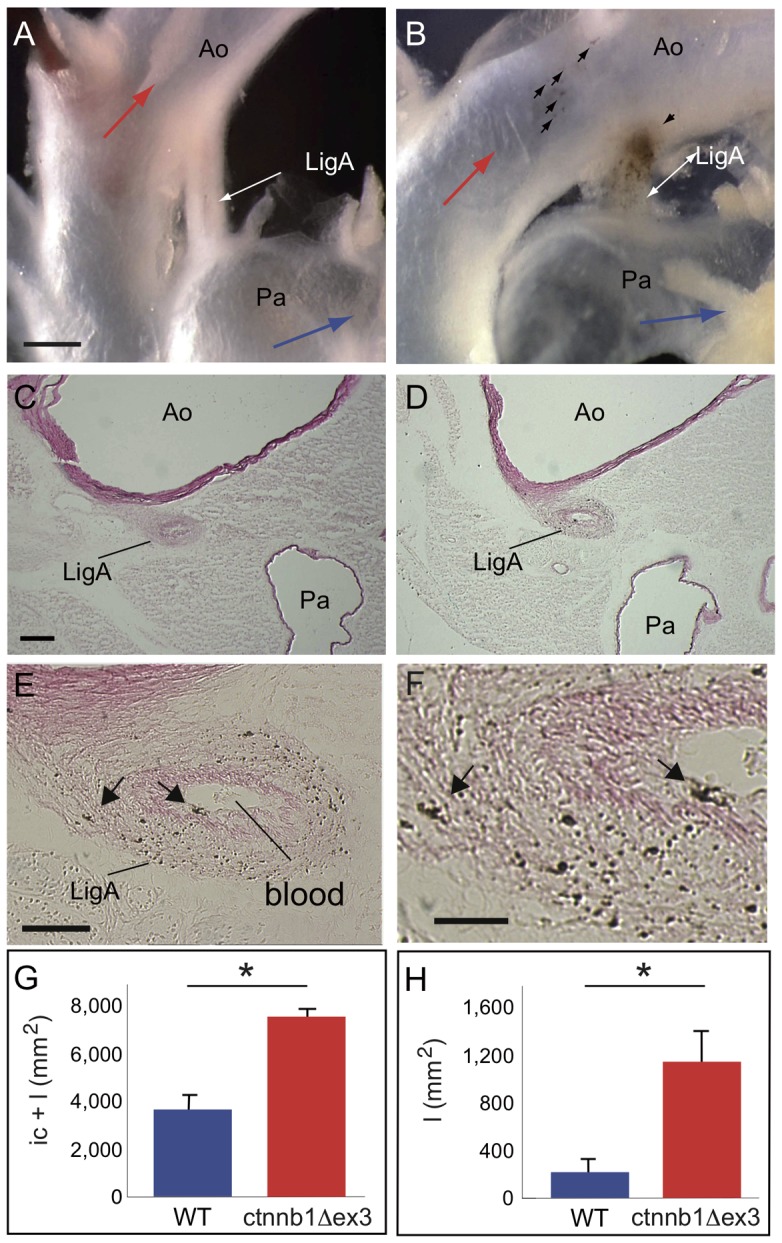
Closure of the ligamentum arteriosum in ctnnb1Δex3 adult heart. The *Tyr::Cre; ctnnb1Δex3*/+ ( =  ctnnb1Δex3) ligamentum arteriosum (LigA) is not fully closed, rendering it a patent ductus arteriosus. At P28, the LigA does not usually show macroscopic hyperpigmentation in wildtype (WT) mice (arrows, A), whereas *Tyr::Cre; ctnnb1Δex3*/+ ( =  ctnnb1Δex3) LigA does (B). Transverse sections show that the WT LigA (C) is fully closed and does not contain any Mc, whereas ctnnb1Δex3 LigA (D–F) is only partially closed, containing both blood in the lumen and numerous pigmented melanocytes in the wall (E, F). The areas occupied by the intimal cushion (ic) and lumen (l) are shown in G and H, respectively. Cross-sections of the LigA show that the outer tunica is dense, while the inner ellipsoid part, known as the intimal cushion, has a distinct aspect. In ctnnb1Δex3 mice, a lumen is observable inside the ic. Ao: aorta, LigA: ligamentum arteriosum, Pa: pulmonary artery, Mc: melanocyte. Scale bars, (A, B)  = 0.25 mm, (C, D)  = 100 µm, (E)  = 50 µm and (F)  = 20 µm. For each genotype, the number of cells were estimated from 8-10 sections per LigA using 4 mice. *: p-value <0.05.

**Figure 12:**
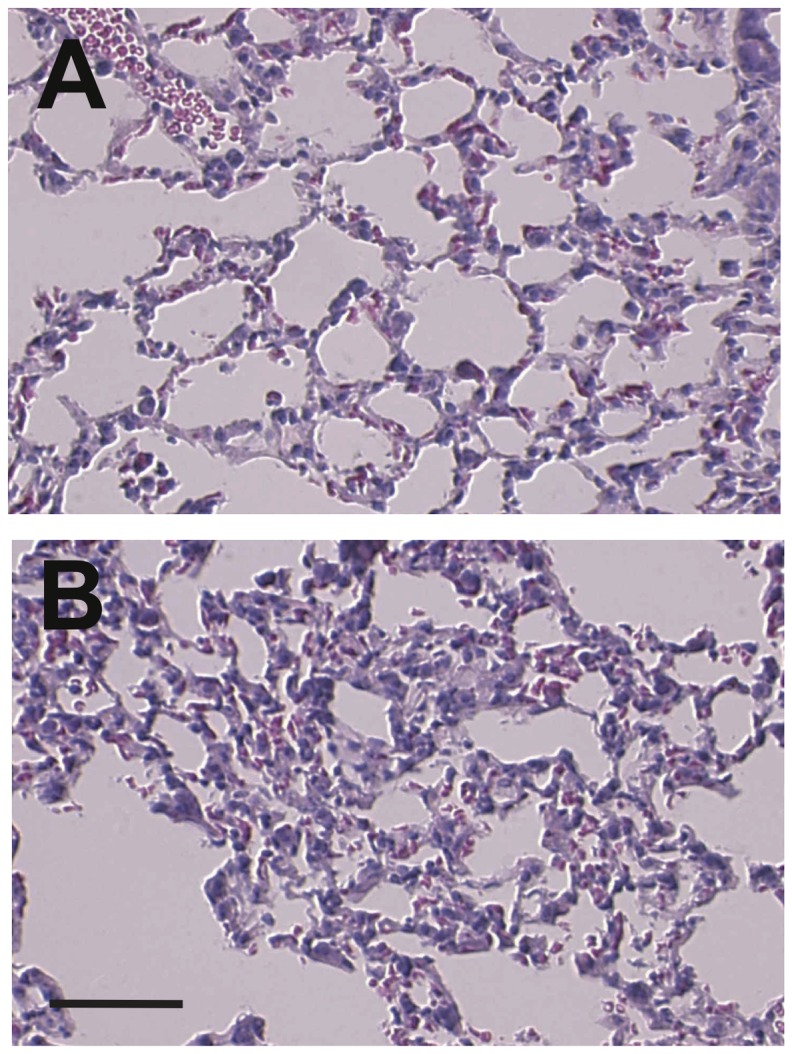
Histological analysis of WT and ctnnb1Δex3 lungs at P28. (A) WT ( = *Tyr::Cre*/°; +/+) and (B) ctnnb1Δex3 mice. Note the disorganized alveolae of the mutant lung. Nonetheless, the lung cells do not express the ctnnb1Δex3 transgene, suggesting that the effect is cell non-autonomous. Scale bars, (A, C, D)  = 50 µm, (B)  = 20 µm.

After birth, the very few Mc in the DA normally differentiate, produce melanin and remain in the LigA. The number of pigmented cells was substantially higher in ctnnb1Δex3 LigA than WT LigA by four weeks (**Figure** **11A, B**). Histological analysis revealed that the ctnnb1Δex3 LigA was not fully closed (**Figure** **11C–F**). The surface areas of the intimal cushion and lumen, when present, were larger in ctnnb1Δex3 than WT mice (**Figure** **11G, H**). Moreover, blood was observed in the canal and large numbers of pigmented cells were present in the tunica media of the ctnnb1Δex3 LigA (**Figures** **11E, F**). Only ctnnb1Δex3 mice presented a PDA, as well as disorganized, fibrotic lung alveolar structures (**Figure** **12**). These observations suggested that abnormally high pulmonary pressure may have been the result of the PDA and cause lung damage in ctnnb1Δex3 mice as compared to WT mice.

### *ctnnb1Δex3* mice can be partially rescued by indomethacin

**Figure 13:**
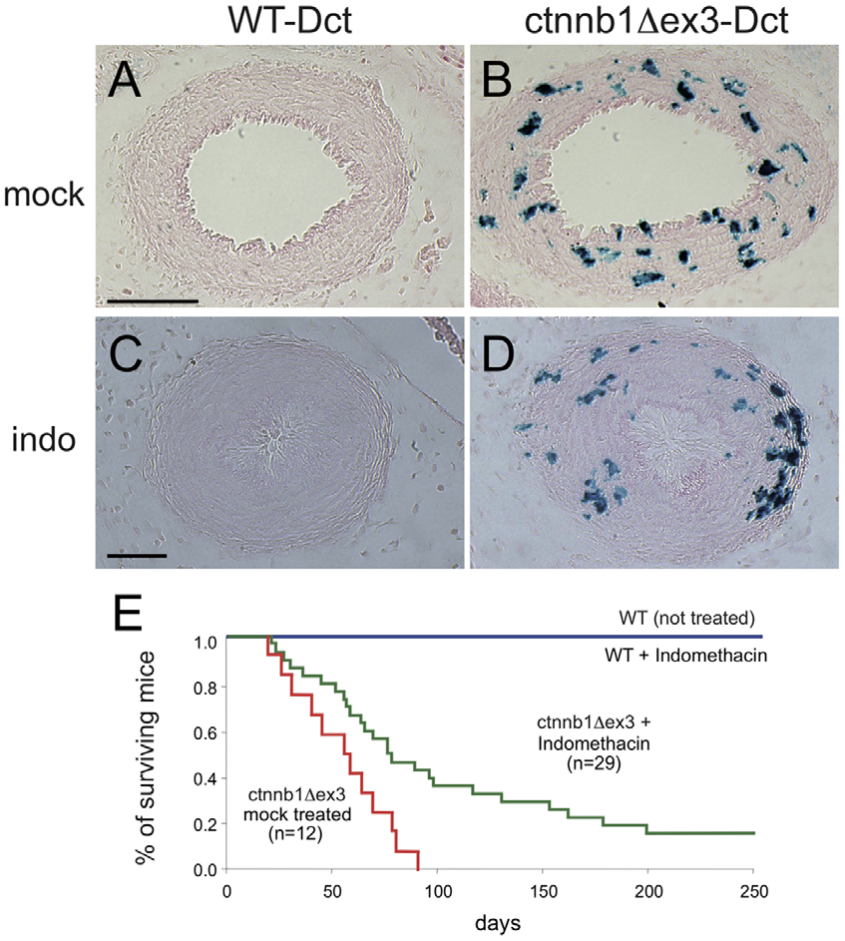
Indomethacin treatment and survival of ctnnb1Δex3 mice. Indomethacin treatment results in the closure of WT and ctnnb1Δex3 ( = *Tyr::Cre/°; ctnnb1Δex3*/+) DA and allows the survival of ctnnb1Δex3 mice. Mock (A, B) and indomethacin (indo, 10 mg/kg body weight) (C, D) intraperitoneal injections into pregnant *Tyr::Cre/Tyr::Cre*; +/+; *Dct::LacZ/Dct::LacZ* females carrying *Tyr::Cre/*°; +/+; *Dct::LacZ/*° (A, C) and *Tyr::Cre/*°; *ctnnb1Δex3*/+; *Dct::LacZ/*° (B, D) E18.5 embryos. Four hours later, embryos were isolated, fixed, X-gal stained, transversally sectioned through the DA and counterstained with eosin. We treated three pregnant females and sectioned ten embryonic hearts (five WT and five mutants). The ductus arteriosus was closed in all cases. Note that the numbers of Dct+ cells derived from ctnnb1Δex3-Dct embryos obtained from pregnant mothers injected with indomethacin or mock-injected were similar. (E) Kaplan-Meier curves of WT and ctnnb1Δex3 newborn pups treated or mock-treated with indomethacin (6 mg/kg body weight indomethacin within 12 hours of birth). Ultrasound analysis was performed on treated versus non-treated animals during the second and third months, which associated survival of treated ctnnb1Δex3 to the size of the left atrium (not shown). Indomethacin-treated ctnnb1Δex3 mice survived significantly longer than mock-treated mice (p<0.009). Note similar results were obtained when ctnnb1Δex3 mi mice were treated with indomethacin or mock. Scale bars, (A, B)  = 100 µm, (C, D)  = 50 µm.

In humans, indomethacin is widely used to treat PDA, by inhibiting the cyclooxygenases that participate in prostaglandin biosynthesis. As a proof of concept, pregnant *Tyr::Cre*/*Tyr::Cre*; +/+; *Dct::LacZ*/*Dct::LacZ* females that had been crossed to *ctnnb1Δex3*/+ males, and thus carrying litters with both WT-Dct and ctnnb1Δex3-Dct embryos, were injected with indomethacin at E18.5 and compared with mock-injected controls. Embryos were removed from three indomethacin-injected females, 4 hours after injection. Five WT-Dct and five ctnnb1Δex3-Dct hearts were isolated and fixed to obtain transverse sections of the DA. Mb were visualized by the activity of β-galactosidase (**Figure** **13A–D**). Indomethacin induced the closure of the DA as expected, in WT-Dct mice, but also by means of the remaining SMC in ctnnb1Δex3-Dct DA.

Newborn ctnnb1Δex3 pups were also directly subcutaneously injected with indomethacin at birth, and this significantly improved their survival rate and indeed cured about twenty per cent of animals (n = 29; **Figure** **13E**). Therefore, ctnnb1Δex3 mice could be partially rescued by indomethacin treatment, suggesting that one of the primary causes of death in ctnnb1Δex3 mice was the failure of full DA closure at birth. However, we cannot exclude the possibility that the left atrium is structurally abnormal in ctnnb1Δex3 mutants, possibly contributing to the death of the animals.

### β-catenin does not lead to overall Cox-2 induction in the DA

β-catenin can directly induce the expression of *Ptgs2* (encoding cyclooxygenase-2 or Cox-2) and stabilizes its mRNA by interacting with AU-rich elements of the 3′-UTR *in vitro* [33], [34]. Cox-2 in turn is known to catalyze the formation of PGE2, and elevated levels of PGE2 are associated with an open DA.

**Figure 14:**
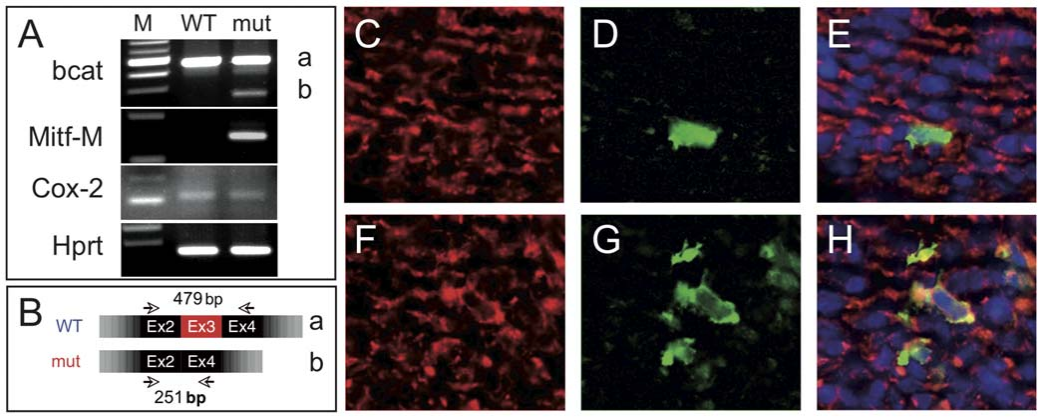
ctnnb1Δex3 is produced in melanoblasts and SMC cells of E18.5 DA. (A) The expression of β-catenin (*bcat*), *Mitf-M* (melanoblasts) and *Hprt* (loading control) was analyzed by RT-PCR on mRNA isolated from WT ( =  Tyr::Cre/°; +/+) and mut ( =  ctnnb1Δex3  =  Tyr::Cre/°; f3/+) DA at E18.5. M corresponds to the size marker. The “a” band (479 bp) corresponds to the non-recombined β-catenin cDNA or WT, whereas the “b” band (251 bp) corresponds to the recombined β-catenin cDNA or ctnnb1Δex3. *Ptgs2* (*Cox2*) is weakly expressed in WT and mutant DA. (B) Schematic of the WT versus mut *bcat* amplicons. (C–H) Immunolocalization of β-catenin in red (C, E, F, H), β-galactosidase in green (D, E, G, H) and DAPI in blue (E, H) in WT-Dct (C–E) and ctnnb1Δex3-Dct (F–H) sections of E18.5 DA. Note that β-catenin is found in both the cytoplasm and the nucleus of ctnnb1Δex3-Dct DA.

One possible explanation for the failure of DA closure in ctnnb1Δex3 mice is that unduly high levels of Cox-2 were induced by the augmented β-catenin signaling. Therefore, we verified that Cre-recombined, truncated ctnnb1Δex3 β-catenin mRNA was detectable in ctnnb1Δex3 DA but not in WT DA, as determined by extracting total RNA from DAs and performing semi-quantitative RT-PCR (**Figure** **14A, B**). β-catenin was visible in both cytoplasm and nuclei, as assessed by immunofluorescence, in both ctnnb1Δex3-Dct and WT-Dct Mb; as expected, more Mb were visible in the former on sections (**Figure** **14C–H**). Nonetheless, the amount of *Ptgs2* (*Cox-2*) mRNA in ctnnb1Δex3 DA was similar to that in WT DA (**Figure** **14A**), indicating that increased β-catenin signaling in ctnnb1Δex3 DA did not result in massive induction of Cox-2. Therefore, Cox-2, although a target of β-catenin, is probably not principally involved in the failure of DA closure in mutant mice at birth.

### ctnnb1Δex3-mi mice lacking melanocytes still die of heart failure

**Figure 15:**
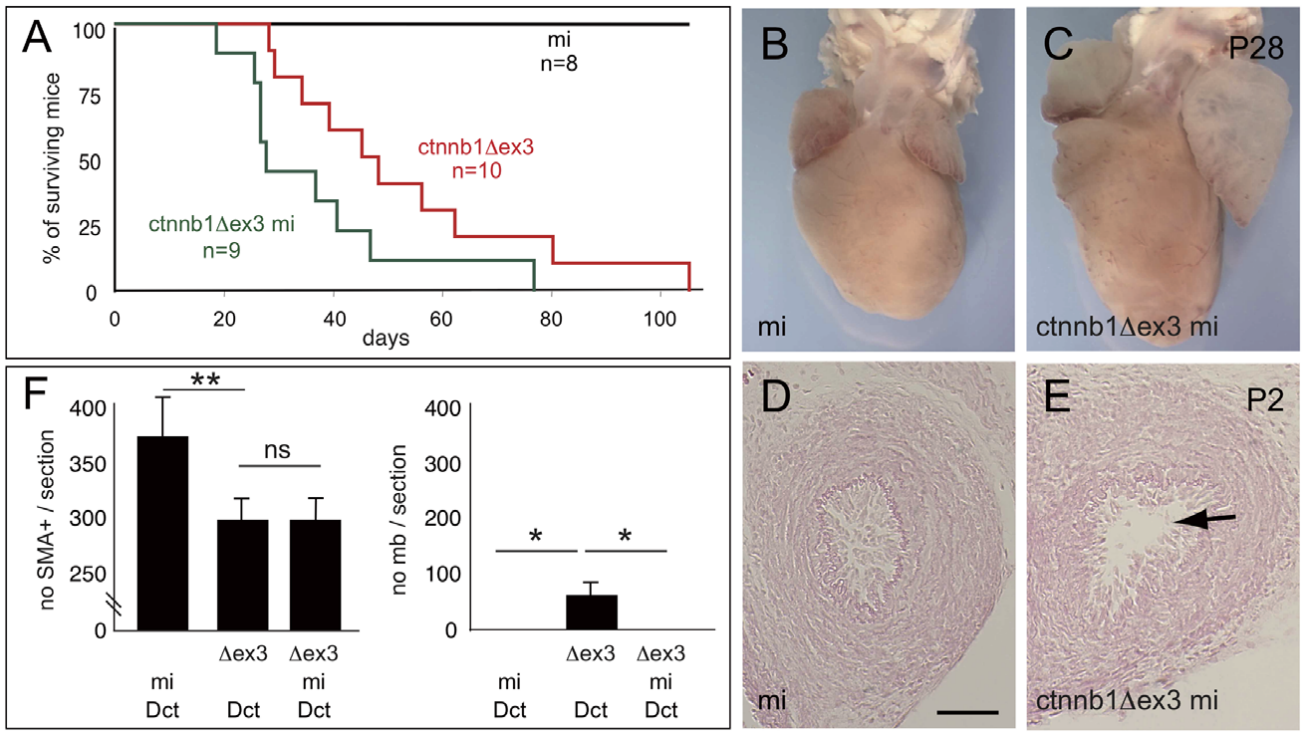
The PDA of ctnnb1Δex3 mice is not rescued by removing melanocytes. (A) Kaplan-Meier survival graph for ctnnb1Δex3-mi ( = *Tyr::Cre/*°; *ctnnb1Δex3/+; mi^vga9^/mi^vga9^*), ctnnb1Δex3 ( = *Tyr::Cre/*°; *ctnnb1Δex3*/+; +/+) and mi ( = *Tyr::Cre*/°; +/+; *mi^vga9^/mi^vga9^*) littermate controls. There is no significant difference between survival of ctnnb1Δex3-mi and ctnnb1Δex3 populations. Macroscopic view of (B) mi and (C) ctnnb1Δex3-mi hearts at P28. Note the enlargement of the ctnnb1Δex3-mi left atrium (*cf*. Figure 8). Transverse eosin-stained sections of (D) mi and (E) ctnnb1Δex3-mi DA at P2. Note that the ctnnb1Δex3-mi DA is not closed (*cf*. Figure 11). (F) Numbers of SMA-positive and LacZ-positive cells were evaluated after SMA, LacZ and DAPI staining in the DA of mi-Dct ( = *Tyr::Cre*/°; +/+; *mi^vga9^/mi^vga9^*; *Dct::LacZ*/°), ctnnb1Δex3-Dct ( = *Tyr::Cre*/°; *ctnnb1Δex3*/+; +/+; *Dct::LacZ*/°) and ctnnb1Δex3-mi-Dct ( = *Tyr::Cre*/°; *ctnnb1Δex3*/+; *mi^vga9^/mi^vga9^*; *Dct::LacZ/*°) mice at E18.5. For each genotype, the number of cells were estimated from 5–10 sections per embryos using 3 mice. mb  =  melanoblast. Scale bar (D, E)  = 50 µm. *: p-value <0.05, **: p-value <0.01, ns  =  non significant.

Mitf-deficient mice (mi  =  *mitf^vga9/vga9^*) have no Mc and a white coat, resulting from a recessive null allele for the microphthalmia-associated transcription factor. In mi mice, Mc are genetically ablated during development at around E11.5, which is after specification of the common SMC2/Mc precursor at about E9.0, but well before birth [22]. To evaluate the contribution of Mc to the full closure of the ctnnb1Δex3 DA at birth, ctnnb1Δex3-mi (*Tyr::Cre*/°; *ctnnb1Δex3/+*; *mitf^vga9/vga9^*) mice were produced. As anticipated, the ctnnb1Δex3-mi mice lacked cutaneous Mc and were white, like mi mice (data not shown). Surprisingly, ctnnb1Δex3-mi mice showed clinical signs similar to ctnnb1Δex3 mice and died at similar ages (**Figure** **15A**). Left atrium enlargement was observed in ctnnb1Δex3-mi mice at P28 (**Figure** **15B, C**). PDA was observed at P2 in ctnnb1Δex3-mi, but not in mi mice (**Figure** **15D, E**).

Additional mice were generated in order to estimate the numbers of Mb, through β-galactosidase activity, and SMC through SMA immunoreactivity, in transverse sections of the DA. *Tyr::Cre*/°; *ctnnb1Δex3*/+; *mi^vga9/vga9^*; *Dct::LacZ*/° (ctnnb1Δex3-mi-Dct) and *Tyr::Cre*/°; +/+; *mi^vga9/vga9^*; *Dct::LacZ*/° (mi-Dct) mice were compared to ctnnb1Δex3-Dct mice (cf. **Figures** **2** and **3**). No Mb were observed in the DA of ctnnb1Δex3-mi-Dct or mi-Dct, in contrast to the ctnnb1Δex3-Dct DA. However, the numbers of SMC were similar in ctnnb1Δex3-Dct and ctnnb1Δex3-mi-Dct DA and reduced relative to the DA of mi-Dct, such that no cellular compensation for SMC was observed in ctnnb1Δex3-mi-Dct DA (**Figure** **15F**).

The general heart morphology appears disrupted in ctnnb1Δex3-mi mice compared to mi mice (**Figure** **15B, C**), as this is the case for ctnnb1Δex3 mice compared to WT mice (**Figure** **7C, D**). A conjunction of events may explain these phenomenona, which are potentially linked, but are not yet understood. These abnormalities could be due to the general blood flow defect in the mutant mice, leading to abnormal pressures on the chambers, thus affecting the morphology. However, it could be associated with melanocytes. Indeed, Mc are present in the heart, and not only in the DA [25], [26]. A careful analysis allowed the visualization of a general increase in Mc numbers in different parts of ctnnb1Δex3 hearts. It includes the foramen ovale (**Figure** **10E**), and the mitral, tricuspid and aortic valves (not shown). In wild-type and ctnnb1Δex3 hearts, no Mc was found in the pulmonary valves. All ctnnb1Δex3 Mc of the heart would have a different expression pattern compared with WT Mc, leading to molecular and cellular modifications. Besides the consequence on the DA, these modifications do not appear dramatic on ctnnb1Δex3 heart. The valves did not present major defects and the Doppler mitral inflow pattern was normal. However, mi mice do not have Mc in the heart due to the lack of Mitf. Mitf is not only expressed in Mc, it is also expressed in cardiomyocytes and is important to regulate cardiac growth and hypertrophy [35]. On C57BL/6 background, mi hearts are not dramatically affected. The conjunction of the lack of Mitf in the heart with the disappearance of an increased number of Mc may explain the different heart morphology and a potential earlier death of ctnnb1Δex3-mi mice, although this hypothesis remains elusive.

In order to confirm that β-catenin does not play a major role in the cardiac Mc lineage after its segregation from SMC2, *Dct::Cre*/°; *ctnnb1Δex3*/+ mice were also produced, and found to be normally viable, with no evident enlargement of the left atrium when sacrificed at one-year old. In these mice, the activated β-catenin is produced specifically in Mb after specification. In another approach, *Tyr::CreERt2*/° mice [36] were crossed with *ctnnb1Δex3*/+ mice to produce a tamoxifen-inducible CreERt2 in *Tyr*-expressing cells. Tamoxifen was administered at E18.5, well after the segregation of the SMC2 and Mc lineages, and all double heterozygous mice were phenotypically normal: viable, again with no enlargement of the left atrium. We counted Dct/Trp2-positive cells, corresponding to Mc, in DA sections at P2 in both tamoxifen- and mock-induced, unrecombined *Tyr::CreERt2*/°; *ctnnb1Δex3*/+ mice. The numbers were very small in both cases, similar to that in WT mice (not shown). Repeating these experiments in both WT and ctnnb1Δex3 backgrounds using mi mice, as expected from the results in **Figure** **15F**, failed to detect any Dct/Trp2-positive cells. The mice were viable and with no cardiac defect.

In conclusion, the DA of ctnnb1Δex3-mi and ctnnb1Δex3 mice did not fully close, demonstrating that the presence or absence of differentiated Mc was irrelevant to the onset of PDA. Rather, the absence of a significant proportion of SMC (SMC2 population) was associated in both cases with the failure of full DA closure and death; indomethacin injection reduced or prevented these manifestations (see **Figure** **13**).

## Discussion

We have demonstrated here that the increased β-catenin activity modifies the fate of a fraction of the smooth muscle cells (SMC2) of the DA into Mc, leading to postnatal PDA. We showed that the presence of numerous Mc in the DA is not the primary cause of the failure of the DA to close. Instead, the substantial reduction in number of SMC2 prevents the closure. ctnnb1Δex3 mice die within a few months, unlike other mutant mice presenting a PDA, which die in the first days after birth.

### SMC2 and Mc have a common precursor

β-catenin can favor the specification of Mb, but it does not induce Mc proliferation [30], [37]. However, β-catenin has been reported to induce cell proliferation of SMC [38], [39]. If SMC2 and Mc had two different precursors, we would expect to observe more SMC2, rather than an increased proportion of Mc in our model system. Conversely, if SMC2 and Mc shared a common precursor, we would expect fewer (or no) SMC2 and an increase in the number of Mc. We observed this latter scenario. The production of more Mc seems to occur at the expense of SMC2 in ctnnb1Δex3 mice. Indeed, we showed that the number of SMC2 missing in the mutant DA was the same as the number of Mb appearing (see **Figure** **5**). Moreover, the same disappearance of SMC2 was observed in mi mice, in which Mc disappear after specification (See **Figure** **15**). These observations suggest that SMC2 and Mc have a common precursor.

However, we cannot formally exclude the existence of other mechanisms, and that the disappearance of SMC2 and presence of high numbers of Mc are not directly linked. For instance, invasion of the DA by Mb may induce the loss of the SMC2 population by an unknown mechanism.

### Absence of SMC2 leads to PDA

The ctnnb1Δex3 mice display incomplete closure of the DA, an enlarged left atrium and die several weeks after birth. The death of these animals is certainly related to thrombus formation. Mc are found in the heart including the left atrium [22], [24], [25], [26], [40]. The enlargement of the left atrium may theoretically be an indirect consequence of an abnormality in the left atrium itself, due to the increased Wnt signaling in Mc, which may affect the expression of various genes and/or have haemodynamic effects. The likelihood of this hypothesis was small considering that ctnnb1Δex3-mi mice do not have Mc in the heart but present the same phenotype as the ctnnb1Δex3 mice. However, we cannot exclude the possibility that these mutant mice (with or without Mc) may present undetected weaknesses of the left atrium *per se*.

Nonetheless, to examine a possible effect from within the differentiated Mc lineage, we conducted some complementary experiments to assess these possibilities. For example, inducing the production of the activated β-catenin within *Tyr*-expressing cells, after the segregation of SMC2 from their common precursors with Mc (at E18.5), yielded a normal cardiac phenotype, as did the expression of the activated β-catenin in Mb specifically (*Dct*-expressing cells).

These approaches, together with our observations in mi mice, indicate that neither the induction of Wnt signaling in *Tyr*- or *Mitf*-expressing Mb, nor the abnormally large number of *Dct*/*Trp2*-expressing Mb in the ctnnb1Δex3 DA, nor both, are sufficient to induce the cardiac phenotype observed. The main cause of the PDA is presumably associated with the absence of SMC2 from the DA. The SMC2 population therefore prevents, rather that the Mc population favors, the development of PDA.

### An induction of the prostaglandin pathway by the activated beta-catenin is not the primary cause of PDA

Fetal PDA is controlled by many factors, the most important of which are proportionally low fetal blood oxygen partial pressure [41] and cyclooxygenase-mediated products of arachidonic acid metabolism (primarily PGE2 and prostacyclin) [42]. Several mutant mouse lines present post-natal PDA as a result of targeting genes of the prostaglandin pathway, acting on contractility (*Ptgs2*, *Pgdh* and *EP4*).

At the molecular level, β-catenin had been shown to induce *Ptgs2*/*Cox-2* expression [33]. Thus, it could have been argued that in our PDA model, the expression of β-catenin in the differentiated, more numerous Mc could lead to the PDA. However, we did not observe an increase in *Ptgs2/Cox-2* expression level in the ctnnb1Δex3 DA, as shown in **Figure** **14**. Moreover, this hypothesis is unlikely because the ctnnb1Δex3-mi DA does not contain cells producing the activated β-catenin, namely SMC2 and Mc, but still presents a PDA.

### Relevance for human PDA

ctnnb1Δex3 and ctnnb1Δex3-mi mice have a smaller number of SMC in the DA than WT mice, and present a dilated left atrium, with moderate left ventricular enlargement in about half of the mice. They suffer a high mortality rate, and this was due to thrombosis and left atrium rupture, rather than the left ventricular failure observed in the human clinical context. These characteristics are thus not the standard characteristics of human PDA. However, although indomethacin is an effective treatment for PDA due to prematurity in humans, it is less effective in term infants, as was also the case for ctnnb1Δex3 mice.

It had not previously been observed that a reduction of smooth muscle cell numbers was correlated with incomplete DA closure in humans. It could theoretically be possible to look for abnormally low numbers of SMC in the DA of term infants with indomethacin-unresponsive PDA, in particular. However, it is difficult (or indeed impossible) to determine objectively whether the numbers of SMC in DA from such patients differ from normal cardiac-healthy, age-matched individuals. There is substantial variability in the number of SMC in various human DA; furthermore there is no ethically appropriate control for determining reference values for normal human DA. To compound things, a very large cohort of normal and abnormal DA would be necessary to compensate for population variability, given the possible genetic differences and effects of the age of the patients. On the other hand, in C57BL/6 mice (a congenic mouse line) at a defined stage, the number of smooth muscle cells in the DA does not differ between individuals.

In human neonates displaying a PDA, left atrium dilation is only observed when the blood velocity of the shunt is high. In these conditions, the left-atrium-to-aortic-root ratio (LAARR) is greater than 1.7; most PDA patients have a LAARR of 1.5. In ctnnb1Δex3 mice, we found that the LAARR ratio was greater than 5 and in many cases around 8 (data not shown), indicating a very high output through the DA and/or a higher compliance of the atrial tissue than in humans. Most of the mouse strains with a high blood flow PDA die shortly after birth, such that *in vivo* evaluation is very difficult. By inactivating the p45 subunit of the transcription factor Nfe2, Echtler et al. [43] studied the contribution of platelets to DA closure in mice. In their homozygous mouse model, they report a lack of DA closure with high blood flow through the channel; they estimated that about 40% of the cardiac output passes through the PDA. These animals show typical features of pulmonary remodeling (in the network of collagen fibers along vessels) and an increase in right ventricular pressure (augmentation of right ventricular wall thickness). Nevertheless, macroscopic analysis of left ventricular sections did not reveal either dilation or hypertrophy. The absence of left ventricular dilation in cases of high flow PDA may be a particular aspect of mouse physiology.

Infants with PDA who are not sufficiently responsive to indomethacin treatment may have an unidentified molecular cause. However, the physiological read-out could be a previously unsuspected cellular defect.

### SMC1 cannot naturally compensate for the lack of SMC2

The main cell components of the tunica media of the DA are the SMC1 and SMC2 populations, which are involved in its closure. In ctnnb1Δex3 mice, the number of SMC2 was reduced in favor of Mc, but although the number of SMC1 remained normal, this was not sufficient to allow the DA to close fully. It is surprising that the SMC1, although numerous, were unable to allow full closure through some sort of compensation mechanism, such as proliferation, in the absence of SMC2. Steric constraints were not imposed by the presence of Mc because, in Mc-deficient ctnnb1Δex3-mi mice, the number of SMC1 remained unaffected and the DA still did not fully close. This latter observation even suggested that the number of SMC1 cell divisions is controlled during development independently of the number of SMC2 cells.

The failure of DA closure associated with the absence of SMC2 could be due to the low total SMC count, or to SMC2 having an essential role in the development of the mature DA (despite SMC1 and SMC2 being histologically indistinguishable). Other differences between SMC1 and SMC2 have not been described. It would be valuable to characterize these two cell types at the molecular level to resolve this question. One possibility could be to look at MYH11 expression, which has been shown to be fully specific of SMC [44]. Potentially, the production/localization of MYH11 could be different in SMC1 and SMC2.

In principle, compensation could occur either at the cellular level, as indicated above, or at a molecular level. Thus, for SMC located in the DA, numerous proteins could contribute to molecular compensation. For instance, a major decrease of Cox-2 production in mutant SMC1 could allow the closure of the DA, although we found no evidence for such a reduction in Cox-2 levels. Other limited molecular compensations may have taken place in SMC1, which would not be sufficient to prevent PDA.

SMC1 and SMC2 may also both be involved in the closure of the DA, but fulfilling different functions. This scenario is consistent with the partial closure of the DA observed in mutant mice where there is about 80% of the WT number of SMC (SMC1). It is nevertheless surprising that the absence of only 20% of the cells (SMC2) is fatal. Hyperstimulation of SMC1 with indomethacin in late gestation ctnnb1Δex3 embryos showed that SMC1 do retain the capacity to close the DA. Moreover, indomethacin treatment of ctnnb1Δex3 newborns with PDA prolonged their life span and even cured some of the animals. Ultrasound analysis revealed that in the cured animals, both the DA and the foramen ovale were closed, and the left atrium was of normal size. However, in the partially cured mice, although the DA was closed, the foramen ovale remained open, which correlates with their enlarged left atria. These differences could be related to the timing of injection/closure of the DA. Another explanation could be related to the increased number of melanocytes in the ctnnb1Δex3 foramen ovale, which might interfere with its closure (**Figure** **10E**).

### Function of melanocytes in the DA and in the heart

Melanocytes are consistently found in all heart valves except pulmonary valves [26]. Moreover, a few Mc are also consistently found in the DA. These observations argue that the presence of Mc in the heart is regulated rather than accidental. Nevertheless, the function of these cardiac Mc remains poorly understood, as these cells do not appear to be essential for general cardiac morphogenesis and physiology. Indeed, mice lacking Mc (e.g. Kit^W/Wv^ or Mitf^vga9/vga9^ mice) or displaying strong pigmentation (e.g. Tyr::Nras^Q61K^) in cardiac valves and septa are viable, with apparently normal hearts [26]. Moreover, we report here that the lack of either pigmented or unpigmented differentiatied melanocytes in the DA does not lead to an observable phenotype. Thus, cardiac Mc would most likely play a subtle role, which would become critical under stress conditions.

Cardiac Mc could be involved in atrioventricular (AV) valves development from the endocardial cushions. Valve development requires remodeling of the extra-cellular matrix [45] and it has been shown that classic Mc produce and secrete metalloproteases [46]. This function of the Mc in the AV valves could continue into adulthood. Indeed, Mc and interstitial cells could be involved in tissue homeostasis and could affect the mechanic properties of the AV valves, as it has been shown that pigmentation stiffens murine tricuspid valve leaflet [47].

Mc of the atrium and pulmonary veins have been shown to contribute to atrial arrhythmia triggers [40]. Deregulation of intracellular calcium and reactive oxygen species (ROS) levels have been described in patients with atrial fibrillation. Dct participates to the regulation of these levels in Mc and mice lacking cardiac Mc do not develop arrhythmias. As we know that skin Mc have a buffer role for various UV-induced physical and chemical stresses, we could hypothesise that cardiac Mc could play a buffer role for calcium and ROS–induced stresses.

The function of the Mc of the DA remains even more elusive, since we showed here that they do not play a role *per se* in the closure of the DA. Their persistence in the LigA after birth suggests that they could maybe play a role in the homeostasis of the region. In zebrafish, numerous Mc have been described surrounding the aorta in the adult, whereas they were absent during development, suggesting a later role [48]. To sum up, it is still not possible to draw conclusions about the physiological role, if any, of Mc in the DA in a healthy and non-stressful environment. However, another descendant from the same precursor, the VNCC-derived SMC2 population, is irreplaceable in preventing PDA by promoting closure of the DA at birth.

## Materials and Methods

### Mice

Crosses are summarized in **Table** **S1**. *Tyr::Cre*, *Tyr::CreERt2*, *Dct::Cre*, β-catenin *ctnnb1Δex3* (also known as f3), β-catenin *ctnnb1Δex2-Δex6* (also known as f2-6), *Dct::LacZ*, *Rosa26R*, and *Mitf^vga9^* mice were used in this study [27], [31], [36], [49], [50], [51], [52], [53], [54]. *Tyr::Cre* mice produce the enzyme Cre, which recognizes and intramolecularly recombines LoxP sequences in cells expressing the tyrosinase promoter at any point throughout life. In *Dct::LacZ* reporter mice, the transgene *LacZ*, producing the enzyme β-galactosidase, is under the control of the dopachrome tautomerase (*Dct*) promoter. *Rosa26R* floxed reporter mice activate the *LacZ* transgene to produce β-galactosidase only within any recombined cells and all of their descendants. This work was carried out in accordance with the Policies of the French Committee of Ethics. Mice were maintained in the SPF mouse colony of the Institut Curie according to French and EU law and is fully accredited by the French Direction of Veterinary Services (C 91 471 108, february 16th, 2011). Animal surgery and experimentation are authorized by the French Direction of Veterinary Services to LL C 91-642, July 20th, 2012) and BF (# 31–205, 2011).

### Mouse embryo and tissue preparation, X-gal staining and immunohistochemistry

Mouse embryos and tissues were collected and rinsed in cold PBS and fixed by incubation for 15–20 minutes in PBS containing 0.25% glutaraldehyde at 4°C. They were rinsed twice in cold PBS and incubated for 16 hours in 30% sucrose/PBS at 4°C, then for 5 hours in sucrose (30%)/OCT(50%)/PBS at 4°C, embedded in OCT compound (TissueTek, 4583, Sakura Finetechnical Co. Ltd. Tokyo, Japan) and sectioned. PBS containing 0.25% glutaraldehyde was injected into the left ventricles of adult mice after anesthesia. Hearts were then removed and treated as described above. For X-gal staining, 8 µm-thick sections were treated as previously described [36]. For immunofluorescence, 8 µm-thick sections were treated as previously described [55]. Whole heart samples placed on agarose gels were observed from the ventral side. All specimens of DA and LigA were sectioned vertically for X-gal staining and immunofluorescence analysis. Antibodies directed against beta-galactosidase and alpha-smooth muscle actin (SMA) were purchased from Abcam (ab9361) and Sigma (A5228), respectively.

### Determination of the intimal cushion (ic) and lumen (l) areas

DA/LigA were sectioned and stained with eosin. The areas of the intimal cushion and the lumen were determined using ImageJ (http://rsbweb.nih.gov/ij/) version 1.37.

### RT-PCR

Total RNA was isolated from mouse LigA with Trizol (Invitrogen). Reverse transcriptase-PCR (RT-PCR) experiments were performed using oligonucleotides specific to β-catenin (*ctnnb1*), Mitf-M (*mitf*) and *Hprt* sequences (**Table** **S2**).

### Echocardiography and micro-CT

For routine examination and color Doppler analysis, transthoracic echocardiograms were performed in a Sonos 5500 on mice anesthetized with 2% isoflurane as described previously (Philips Ultrasound, USA) [56]. To visualize the DA and foramen ovale, high-resolution imaging was performed with a 30 MHz probe on a Vevo 660 (Visualsonics, Canada) with the same anesthetic regimen. Due to the rightward shift of the heart of ctnnb1Δex3-mice, non-conventional incidences were employed. Mice were processed as previously described for micro-CT [57].

### Indomethacin treatment

An indomethacin (Sigma) stock solution was prepared as a 10 mg/ml solution in ethanol and stored at −20°C. Working solutions were prepared extemporaneously as 500 ng/µl solutions in PBS. E18.5 pregnant females were injected intraperitoneally with 10 mg/kg body weight indomethacin and the embryos harvested 4 hours later. Newborn mice were injected subcutaneously with 6 or 12 mg/kg body weight indomethacin within 12 hours of birth, using a microsyringe (Fisher-Bioblock, France W23305). No difference in long-term survival was observed between animals treated with the two doses of indomethacin.

## Supporting Information

Table S1 **Abbreviation, Genotype and main characteristics of the used transgenic animals.** Rosa26R allows to follow up the defloxed cells [53]. Dct::LacZ allows to visualize melanoblasts/melanocytes [52]. Tyr::Cre allows to deflox gene from E9.5 in some vagal neural crest derivatives, in particular melanocytes [22], [27]. Tyr::CreERt2 allows to deflox gene after tamoxifen induction in melanocytes [36]. Dct::Cre allows to deflox gene after E12.5 in a chimeric way in melanocytes [50]. Melanoblasts stop expanding in mi^vga9/vga9^ mice [54]. The β-catenin gene (Ctnnb1) was floxed in the introns 2 and 3 (ctnnb1Δex3) [31].
(DOC)

Table S2 **Oligonucleotides used to determine the presence of β-catenin, Mitf-M and Hprt and the length of the amplicons.**
(DOC)

Movie S1 **Reconstructed truncal regions from CT scan pictures of WT** (***Tyr::Cre/*** **°; +/+**) **at the truncal level at P28.**
(AVI)

Movie S2 **Reconstructed truncal regions from CT scan pictures of ctnnb1Δex3** (***Tyr::Cre/*** **°;** ***ctnnb1Δex3/+***) **at the truncal level at P28.** Note in *Tyr::Cre/*°; *ctnnb1Δex3/+*; mice, the left atrium is so enlarged that it affects the angular orientation of the heart by displacement.
(AVI)
