# Supplementary material for: A Subpopulation of Smooth Muscle Cells, Derived from Melanocyte-Competent Precursors, Prevents Patent Ductus Arteriosus
Source: PLoS One. 2013 Jan 31;8(1):e53183. doi: 10.1371/journal.pone.0053183 (PMC3561373; doi:10.1371/journal.pone.0053183)
Supplement: Table S1 — Abbreviation, Genotype and main characteristics of the used transgenic animals. Rosa26R allows to follow up the defloxed cells [53]. Dct::LacZ allows to visualize melanoblasts/melanocytes [52]. Tyr::Cre allows to deflox gene from E9.5 in some vagal neural crest derivatives, in particular melanocytes [22], [27]. Tyr::CreERt2 allows to deflox gene after tamoxifen induction in melanocytes [36]. Dct::Cre allows to deflox gene after E12.5 in a chimeric way in melanocytes [50]. Melanoblasts stop expanding in mivga9/vga9 mice [54]. The β-catenin gene (Ctnnb1) was floxed in the introns 2 and 3 (ctnnb1Δex3) [31]. (DOC) [file pone.0053183.s001.doc]

Abbreviation Genotype

______________ _________________________________

WT *Tyr::Cre/°; +/+*

ctnnb1Δex3 *Tyr::Cre/°; ctnnb1Δex3/+*

WT-Dct *Tyr::Cre/°; +/+; Dct::LacZ/+*

ctnnb1Δex3-Dct Tyr::Cre/°; *ctnnb1Δex3*/+; Dct::LacZ/+

WT-Rosa *Tyr::Cre/°; +/+; Rosa26R/+*

ctnnb1Δex3-Rosa *Tyr::Cre/°; ctnnb1Δex3/+; Rosa26R/+*

mi *mi^vga9/vga9^*

ctnnb1Δex3-mi *Tyr::Cre/°; ctnnb1Δex3/+; mi^vga9/vga9^*

*Tyr::Cre/°; ctnnb1Δex2-6/+*

*Tyr::Cre/°; ctnnb1Δex2-6/ctnnb1Δex2-6*

*Dct::Cre/°; +/+*

*Dct::Cre/°; ctnnb1Δex3/+*

*Tyr::CreERt2/°;* *mi^vga9/vga9^*

*Tyr::CreERt2/°; ctnnb1Δex3/+;*

*Tyr::CreERt2/°; ctnnb1Δex3/+; mi^vga9/vga9^*

______________ _________________________________________

**Table S1** Yajima et al
