# Supplementary material for: A Subpopulation of Smooth Muscle Cells, Derived from Melanocyte-Competent Precursors, Prevents Patent Ductus Arteriosus
Source: PLoS One. 2013 Jan 31;8(1):e53183. doi: 10.1371/journal.pone.0053183 (PMC3561373; doi:10.1371/journal.pone.0053183)
Supplement: Table S2 — Oligonucleotides used to determine the presence of β-catenin, Mitf-M and Hprt and the length of the amplicons. (DOC) [file pone.0053183.s002.doc]

β-catenin:

Forward : 5’-GTC AGC TCG TGT CCT GTG AA-3’ (LL765)

Reverse : 5’- TCT GTG ATG GTT CAG CCA AG-3’ (LL766)

Size of the amplicon.

WT allele : 566 bp,

defloxed ctnnb1 Δex3 allele 336 bp.

Mitf-M:

Forward : 5’-TGA AAC CTT GCT ATG CTG GA-3’ (LL833)

Reverse 5’-TAC CTG GTG CCT CTGAG CTT-3’ (LL834)

Size of the amplicon : 108 bp.

Hprt:

Forward : 5’-CAC AGG ACT AGA ACA CCT GC-3’ (LL017)

Reverse 5’-GCT GGT GAA AAG GAC CTC T-3’ (LL018)

Size of the amplicon : 249 bp.

**Table S2**

Yajima et al
